# Bupi Yishen Formula Versus Losartan for Non-Diabetic Stage 4 Chronic Kidney Disease: A Randomized Controlled Trial

**DOI:** 10.3389/fphar.2020.627185

**Published:** 2021-01-29

**Authors:** Wei Mao, Nizhi Yang, Lei Zhang, Chuang Li, Yifan Wu, Wenwei Ouyang, Peng Xu, Chuan Zou, Chunpeng Pei, Wei Shi, Jihong Zhan, Hongtao Yang, Hongyu Chen, Xiaoqin Wang, Yun Tian, Fang Yuan, Wei Sun, Guoliang Xiong, Ming Chen, Jianguo Guan, Shuifu Tang, Chunyan Zhang, Yuning Liu, Yueyi Deng, Qizhan Lin, Fuhua Lu, Weihong Hong, Aicheng Yang, Jingai Fang, Jiazhen Rao, Lixin Wang, Kun Bao, Feng Lin, Yuan Xu, Zhaoyu Lu, Guobin Su, La Zhang, David W Johnson, Daixin Zhao, Haijing Hou, Lizhe Fu, Xinfeng Guo, Lihong Yang, Xindong Qin, Zehuai Wen, Xusheng Liu

**Affiliations:** ^1^ Guangdong Provincial Hospital of Chinese Medicine, The Second Affiliated Hospital of Guangzhou University of Chinese Medicine, Guangzhou, China; ^2^ Department of Global Public Health, Karolinska Institute, Stockholm, Sweden; ^3^ The First Affiliated Hospital of Heilongjiang University of Chinese Medicine, Ha’erbin, China; ^4^ The First Affiliated Hospital of Guangxi University of Chinese Medicine, Nanning, China; ^5^ The First Affiliated Hospital of Guiyang University of Traditional Chinese Medicine, Guiyang, China; ^6^ First Teaching Hospital of Tianjin University of Traditional Chinese Medicine, Tianjin, China; ^7^ Hangzhou Hospital of Traditional Chinese Medicine, Hangzhou, China; ^8^ Hubei Province Hospital of Traditional Chinese Medicine, The Affiliated Hospital of Hubei University of Chinese Medicine, Wuhan, China; ^9^ Shanxi Hospital of Traditional Chinese Medicine, Xi’an, China; ^10^ The Affiliated Hospital of Liaoning University of Traditional Chinese Medicine, Shenyang, China; ^11^ Jiangsu Province Hospital of Traditional Chinese Medicine, Nanjing, China; ^12^ Shenzhen Traditional Chinese Medicine Hospital, Shenzhen, China; ^13^ The Teaching Hospital of Chengdu University of Traditional Chinese Medicine, Chengdu, China; ^14^ Liu Zhou Traditional Chinese Medical Hospital, Liuzhou, China; ^15^ The First Affiliated Hospital of Guangdong University of Chinese Medicine, Guangzhou, China; ^16^ Yunnan Provincial Hospital of Traditional Chinese Medicine, Kunming, China; ^17^ Dongzhimen Hospital to Beijing University of Chinese Medicine, Beijing, China; ^18^ Longhua Hospital Affiliated to Shanghai University of Traditional Chinese, Shanghai, China; ^19^ Zhu Hai Hospital of Guangdong Provincial Hospital of Chinese Medicine, Zhuhai, China; ^20^ The Affiliated Jiang men Traditional Chinese Medicine Hospital, Jinan University, Jiangmen, China; ^21^ The First Affiliated Hospital to Shanxi Medical University, Taiyuan, China; ^22^ Guangzhou Hospital of Traditional Chinese Medicine, Guangzhou, China; ^23^ Xinhui Hospital of Traditional Chinese Medicine, Jiangmen, China; ^24^ Royal Melbourne Institute of Technology, Melbourne, VIC, Australia; ^25^ Department of Nephrology, Princess Alexandra Hospital, Brisbane, Australia, University of Queensland, Brisbane, Australia, Translational Research Institute, Brisbane, Australia; ^26^ Guangdong Provincial Hospital of Chinese Medicine, The Second Affiliated Hospital of Guangzhou University of Chinese Medicine, Guangzhou, China; Guangzhou University of Chinese Medicine, Guangzhou, China

**Keywords:** traditional Chinese medicine, losartan, chronic kidney disease, randomized controlled trial, glomerular filtration rate

## Abstract

Chinese herbal medicine (CHM) might have benefits in patients with non-diabetic chronic kidney disease (CKD), but there is a lack of high-quality evidence, especially in CKD4. This study aimed to assess the efficacy and safety of Bupi Yishen Formula (BYF) vs. losartan in patients with non-diabetic CKD4. This trial was a multicenter, double-blind, double-dummy, randomized controlled trial that was carried out from 11-08-2011 to 07-20-2015. Patients were assigned (1:1) to receive either BYF or losartan for 48 weeks. The primary outcome was the change in the slope of the estimated glomerular filtration rate (eGFR) over 48 weeks. The secondary outcomes were the composite of end-stage kidney disease, death, doubling of serum creatinine, stroke, and cardiovascular events. A total of 567 patients were randomized to BYF (*n* = 283) or losartan (*n* = 284); of these, 549 (97%) patients were included in the final analysis. The BYF group had a slower renal function decline particularly prior to 12 weeks over the 48-week duration (between-group mean difference of eGFR slopes: −2.25 ml/min/1.73 m^2^/year, 95% confidence interval [CI]: −4.03,−0.47), and a lower risk of composite outcome of death from any cause, doubling of serum creatinine level, end-stage kidney disease (ESKD), stroke, or cardiovascular events (adjusted hazard ratio = 0.61, 95%CI: 0.44,0.85). No significant between-group differences were observed in the incidence of adverse events. We conclude that BYF might have renoprotective effects among non-diabetic patients with CKD4 in the first 12 weeks and over 48 weeks, but longer follow-up is required to evaluate the long-term effects.

**Clinical Trial Registration:** http://www.chictr.org.cn, identifier ChiCTR-TRC-10001518.

## Introduction

Chronic kidney disease (CKD) is a major global health problem with poor outcomes and high medical costs, especially when patients progress to end-stage kidney disease (ESKD) (GBD Chronic Kidney Disease Collaboration, 2020). In China, the prevalence of CKD ranges from 9.4% to 13.0% (Zhang et al., 2012). Patients with stage 4 CKD accounts for 0.16% of the world's population (GBD Chronic Kidney Disease Collaboration, 2020). The principles of treatment in CKD4 include statins to control blood lipids, low-salt, high-quality, and low-protein diet, and anti-inflammatory drugs (de Zeeuw et al., 2013), but efficacy is low.

The mainstay of medical management to prevent CKD progression is to achieve optimal control of blood pressure (BP) with antihypertensive agents, particularly angiotensin-converting enzyme inhibitors (ACEi) or angiotensin receptor blockers (ARB) (Palmer et al., 2015). Nevertheless, the use of ACEI/ARB is limited because of high potassium (Ahuja et al., 2000) and aggravated renal failure (Tomlinson et al., 2013) in stage 4 CKD. More than half of users discontinued ACEI/ARB within 5 years of therapy initiation (Qiao et al., 2019).

The limitations in managing CKD4 drive numerous patients to seek Chinese herbal medicines (CHMs). Western guidelines advise that herbal supplements should not be used in CKD, partially due to concerns about a lack of supportive clinical evidence (Levin and Stevens, 2014). Nevertheless, CHMs are commonly used worldwide (Eisenberg et al., 1998). In China and other Asian countries, CHMs are widely used in patients with CKD to delay renal failure (Chen et al., 2007). Nearly half (45.3%) of the patients with CKD in Taiwan use CHM (Lin et al., 2015). Although evidence for the renoprotective benefits of CHM has been gradually accumulating (Lin et al., 2015), these studies have mainly focused on patients with early (stage 1–3) CKD or diabetes (Chen et al., 2013; Zhang et al., 2014b; Zhang et al., 2014c), but there are few high-quality studies in CKD4.

The Bupi Yishen Formula (BYF) is a patent CHM originating from the historical Si-jun-zi Decoction. It was developed based on the text mining result of 10,000 medical records from Guangdong Provincial Hospital of Chinese Medicine (GPHCM), a tertiary hospital in southern China. Radix astragali and Salvia miltiorrhiza can delay CKD (Feng et al., 2013; Care, 2017) and are the main components of BYF. BYF is composed of 86 compounds, including flavonoids, saponins, and phenolic acids (Zhang et al., 2018b). A previous study by our group (Li et al., 2013) showed an association between BYF use and preservation of renal function in patients with advanced CKD.

The aim of this multicenter randomized controlled trial (RCT) was to assess the efficacy and safety of BYF in renal progression vs. losartan. The results could help ease the burden of renal failure in patients with CKD4 without diabetes. In China, ARB, especially losartan, is used more frequently than ACEI due to the side effects such as cough and was selected for the control group.

## Materials and Methods

### Study Design

This trial was a multicenter, double-blind, double-dummy, randomized controlled trial that was carried out from November 8, 2011, to July 20, 2015. Twenty-one hospitals in mainland China participated in this trial (Supplementary Table S1). The eligible patients were consecutively enrolled and randomized 1:1 ratio to receive BYF or losartan for 48 weeks.

### Ethics

The study protocol was approved by the Institutional Ethics Committee of GPHCM (approval ref. B2010-11-03) and registered at the Chinese Clinical Trial Registry Number: ChiCTR-TRC-10001518. All participants provided written informed consent before randomization.

### Patients

Detailed inclusion and exclusion criteria for this trial are described in the protocol (Mao et al., 2015). We included eligible non-diabetic adult patients (18–80 years) diagnosed with CKD4 (Hou et al., 2007) for at least 3 months, with well-controlled blood pressure (<140/90 mmHg) and the Chinese Medicine syndrome of Spleen and Kidney Qi deficiency.

### Randomization and Blinding

Randomization was conducted by the personnel from the Key Unit of Methodology in Clinical Research of the GPHCM. Randomization number list was generated using the PROC PLAN procedure in SAS 9.2 (SAS Institute, Cary, NC, United States). The randomization was stratified according to the center with confidential block size. The blinding codes were delivered to each site via a web-based system to conceal allocation.

Patients, investigators, monitors, outcome assessors, and statisticians were blinded. Masking for patients and site personnel was achieved by double-dummy placebo design. The placebos of BYF and losartan were matched to their corresponding drug in appearance, color, and taste.

### BYF and Placebos

The BYF herbs were extracted with hot water, concentrated, spray-dried, made into granules, and packed in sealed opaque sachets. Production was performed by Pura Pharm Pharmaceuticals Co Ltd (Nanning, Guangxi, China) and controlled rigorously according to good manufacturing practice (GMP) standards. The BYF placebo was produced by the same manufacturer as the original using a concentrate of Colyx Tea (leaf of Broadleaf Holly), caramel pigments, gardenia yellow pigment, sunset yellow pigment, saccharose, and dextrin. The BYF placebo was similar in color, smell, taste, appearance, and packaging with the BYF granule. Qualities of BYF and placebo granules, such as appearance, determination of water, size of granule, solubility, hygroscopicity, heavy metals, toxic elements, pesticide residues, and microbial limit, were controlled rigorously according to the 2010 Chinese pharmacopeia and Chinese Medicine Council of Hong Kong.

### Intervention

After enrollment and prior to randomization, the participants who were taking ACEi and/or ARB before enrollment were administered alternative antihypertensive agents during a 3 week run-in period according to the protocol (Mao et al., 2015). In the BYF group, the participants were instructed to dissolve 15 g of BYF granules (Supplementary Tables S2,S3) in 150 ml of boiled water and to take this solution orally thrice daily, and losartan matched placebo tablet once daily for 48 weeks. The participants in the losartan group were instructed to take 100 mg losartan orally once daily along with 15 g of BYF matched placebo thrice daily for 48 weeks. There were no dose adjustments.

All participants received conventional CKD management according to guidelines (Levin and Stevens, 2014). Any ACEi/ARB or CHM (other than the study medication) was prohibited during the trial. Keto-amino acids were originally prohibited. However, as most participants refused to stop keto-amino acids, the protocol was subsequently amended to allow their concurrent use. Compliance was monitored by dose counting.

### Follow-Up

Patients were examined at baseline and at weeks 2, 4, 6, and 8, and thereafter every 4 weeks until 48 weeks. At each visit within the first 8 weeks, serum creatinine and urea nitrogen were measured. Thereafter, urinary protein-to-creatinine ratio, serum creatinine, urea nitrogen, calcium, phosphate, serum albumin, liver enzymes (aspartate aminotransferase and alanine aminotransferase), blood lipids (cholesterol, triglycerides, and low-density lipoprotein cholesterol), intact parathyroid hormone, and blood cell counts were measured at weeks 12, 24, 36, and 48. Serum potassium was examined at each time point for safety monitoring. BP was monitored at home for one week prior to each visit. BP was also measured at each visit, in the sitting position, after a 5 min rest. Two measurements were taken; if they were >10 mmHg apart, a third measurement was taken.

### Outcomes and Definitions

The primary outcome was the difference in the slope of CKD-EPI eGFR (Inker et al., 2012) between the two groups over 48 weeks. The laboratory assays for serum creatinine measurement were standardized across all sites and repeatedly cross-checked to ensure the comparability of eGFR results.

The secondary outcomes were time to the first occurrence of a composite outcome (death from any cause, doubling of serum creatinine level, ESKD, stroke, or cardiovascular events), and time to each of the individual components of the composite outcome. ESKD was defined as eGFR decreasing to <15 ml/min/1.73 m^2^ for >4 weeks, or requiring permanent dialysis or kidney transplantation. Stroke was defined as either ischemic cerebral infarction or cerebral hemorrhage. Cardiovascular events were defined as acute myocardial infarction and heart failure. Urinary protein-to-creatinine ratio, serum creatinine, urea nitrogen, serum albumin, blood lipids profile (cholesterol, triglycerides, and low-density lipoprotein cholesterol), calcium, phosphate, and intact parathyroid hormone were measured as secondary outcomes.

The participants were asked to report any symptoms and adverse events (AEs) at each follow-up visit or immediately when they happened. In addition, serum potassium, liver enzymes of aspartate aminotransferase and alanine aminotransferase, blood cell counts, routine urine and stool tests, and electrocardiography were checked as safety indices.

### Sample Size

Based on the results of previous retrospective data and trial by Hou et al., (2006), a sample size of 221 patients per group was estimated to provide 90% power and a one-sided significance level of 2.5% to detect a between-group difference of mean eGFR change of 2.3 ml/min/1.73 m^2^ per year with a superiority test, assuming a standard deviation of 4.2 and a clinically important difference of 1 ml/min/1.73 m^2^. The final sample size was adjusted to 554 assuming 20% dropout.

### Statistical Analysis

The primary outcome was analysed according to the Intent-to-Treat (ITT) principle using the full analysis set (FAS), i.e. all treated patients with a baseline eGFR and at least one eGFR value on treatment. Safety analyses were performed on the safety set (SS). The change of eGFR at week 48 from baseline was analyzed by fitting a mixed-effects model in which treatment and the interaction of treatment and time were treated as a fixed effect while the time effect as a random effect.

Randomization should mitigate differences in characteristics between the two groups, but because of missing data, the mixed-effects model was used instead of repeated measure ANOVA, and adjustments were made for the sake of conservativeness and insurance.

The log-rank test was used to compare time to composite outcome between the two groups, followed by unadjusted and adjusted Cox proportional hazards model estimations. Other continuous outcomes were assessed using the *t*-test or Mann-Whitney U-test, as appropriate. Categorical variables were compared using Fisher’s exact test or Mann-Whitney U-test, as appropriate. Subgroup analyses were repeated in strata according to sex, age (≤45 years or >45 years), hypertension, and hyperuricemia.

Sensitivity analyses were conducted: 1) To test the robust of the primary analysis, a mixed-effects model using baseline eGFR, sex, age, mean change of weight, history of hypertension, and history of gout or hyperuricemia as covariates; 2) Missing data on the primary outcome were imputed using the multiple imputation method under the missing at random assumption. 3) to detect whether there was a change in slope before and after 12 weeks, a piecewise linear mixed-effects model was used; 4) using different methods of imputing missing outcome data: multiple imputations and mixed model for repeated measures; 5) excluding patients who experienced a serum creatinine increase more than 30% within the first 12 weeks; and 6) excluding patients who took keto-amino acids during the trial.

All analyses were performed using the PASS 18.0 (IBM Corporation, Armonk, New York, USA) and STATA 11.0 (Stata Corp. College Station, Texas, USA) with a two-sided *p*-value <0.05 considered statistically significant.

## Results

### Patients’ Characteristics

**FIGURE 1:**
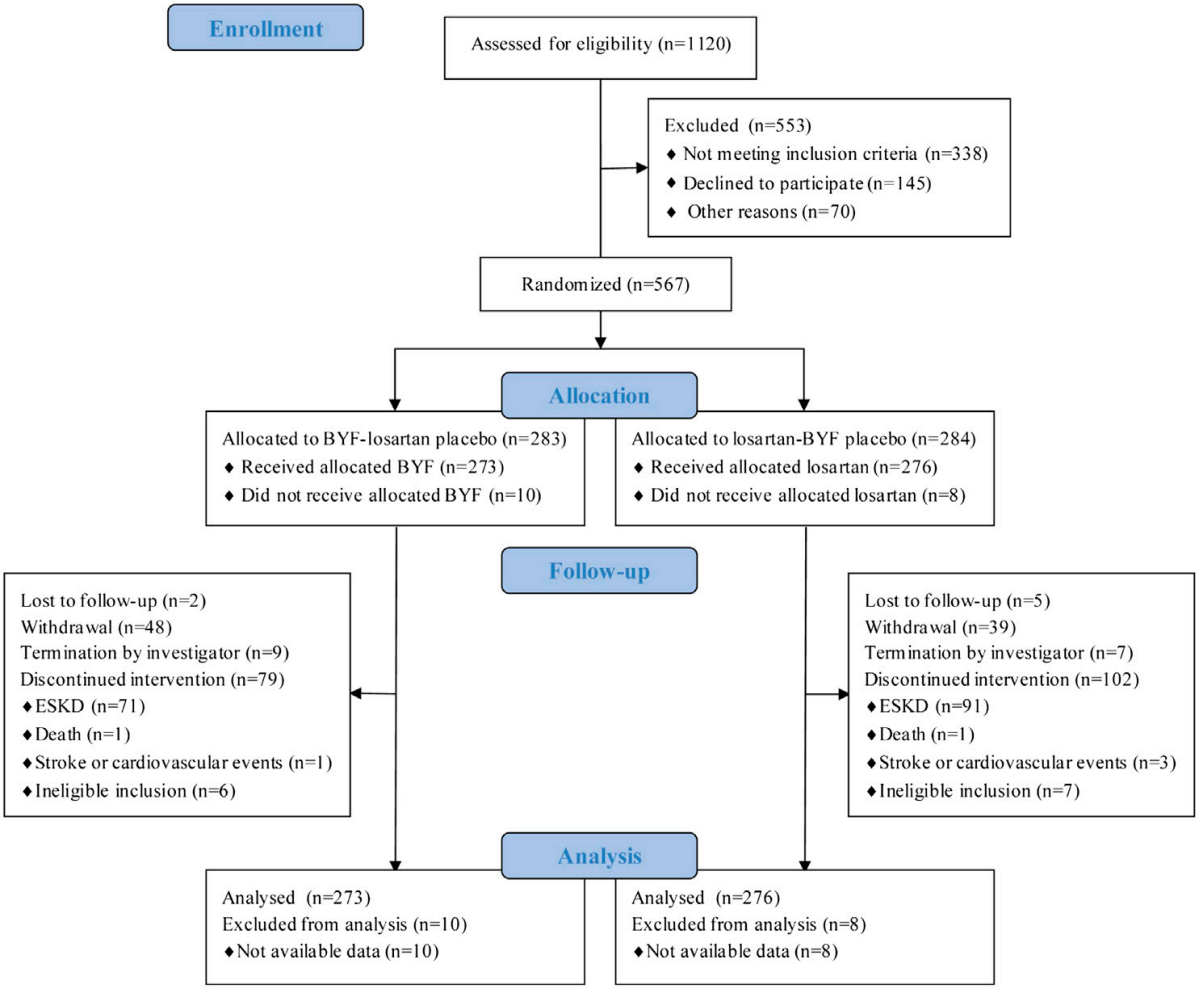
Participant flowchart.

A total of 1120 patients were screened at 21 centers; 567 agreed to participate and were randomized to BYF (*n* = 283) or losartan (*n* = 284) (Figure 1); 549 participants were included in the FAS since 18 participants were excluded due to no available post-baseline data. The compliance with study medication was 90.1% (246/273) in the BYF group and 94.6% (261/276) in the losartan group. The median follow-up was 47.4 weeks.

The baseline characteristics are shown in Table 1. The mean age was 52.2 ± 14.1 years, 250 (44.1%) were female, and 529 (93.3%) were of Han ethnicity. The most common primary renal disease was chronic glomerulonephritis (377 participants, 66.5%), and the mean eGFR at baseline was 22.4±5.8 ml/min/1.73 m^2^. During the run-in period, antihypertensive drugs except for ACEi and ARB were prescribed to control BP. Of 567 participants, only 23 used ACEi or ARB before enrollment and were shifted to other types of antihypertensive drugs.

**TABLE 1:** Baseline characteristics of the study participants (FAS population).

Characteristics	Total (*n* = 567)	Losartan group (*n* = 284)	BYF group (*n* = 283)
Male sex, *n* (%)	317 (55.9%)	151 (53.2%)	166 (58.7%)
Age (years), mean ± SD	52.2 ± 14.1	51.9 ± 14.1	52.5 ± 14.0
Ethic original, *n* (%)			
Han ethnicity	529 (93.3%)	265 (93.3%)	264 (93.3%)
Other ethnicities	38 (6.7%)	19 (6.7%)	19 (6.7%)
Body mass index (kg/m^2^)^a^, mean±SD	22.9 ± 3.4	22.9 **±** 3.3	22.9 **±** 3.5
Primary renal disease, *n* (%)			
Chronic glomerulonephritis	377 (66.5%)	189 (66.5%)	188 (66.5%)
Polycystic kidney disease	14 (2.5%)	8 (2.8%)	6 (2.1%)
Obstructive nephropathy	15 (2.6%)	8 (2.8%)	7 (2.5%)
Other	161 (28.4%)	79 (27.9%)	82 (29.0%)
Estimated GFR (ml/min/1.73 m^2^) ^b^, mean ± SD	22.4 ± 5.8	22.3 ± 5.5	22.4 ± 5.4
Serum creatinine (µmol/L)^c^, mean ± SD	255.8 ± 66.7	253.5 **±** 55.8	258.1 **±** 76
Urinary protein-to-creatinine ratio^d^, median (IQR)	1.3 (0.6,2.2)	1.4 (0.6, 2.3)	1.3 (0.7, 2.1)
Blood pressure (mm Hg), mean ± SD			
Systolic	128.0 ± 9.4	127.5 **±** 8.8	128.5 **±** 10.0
Diastolic	79.7 ± 7.3	79.4 **±** 7.4	80.1 **±** 7.2
Hypertension history, *n* (%)	296 (52.2%)	147 (51.8%)	149 (52.7%)
ACEi/ARB medication history, *n* (%)	23 (4.2%)	12 (4.3%)	11 (4.0%)
Gout/hyperuricemia history, *n* (%)	90 (15.9%)	43 (15.1%)	47 (16.6%)
Keto-amino acids medication, *n* (%)	40 (7.3%)	25 (9.1%)	15 (5.5%)
Alfacalcidol medication, *n* (%)	22 (4.0%)	10 (3.6%)	12 (4.4%)

^a^ Calculated as weight in kilograms divided by height in meters squared.

^b^ Two participants in the losartan group had no available data of baseline eGFR.

^c^ Two participants in the losartan group had no available data of baseline serum creatinine.

^d^ 111 patients in the losartan group and 102 participants in the BYF group had no available data of baseline urinary protein-to-creatinine ratio.

BYF, Bupi Yishen Formula; SD, standard deviation; GFR, glomerular filtration rate; IQR, interquartile range; ACEi, angiotensin-converting enzyme inhibitor; ARB, angiotensin receptor blocker.

### Primary Outcome

**FIGURE 2:**
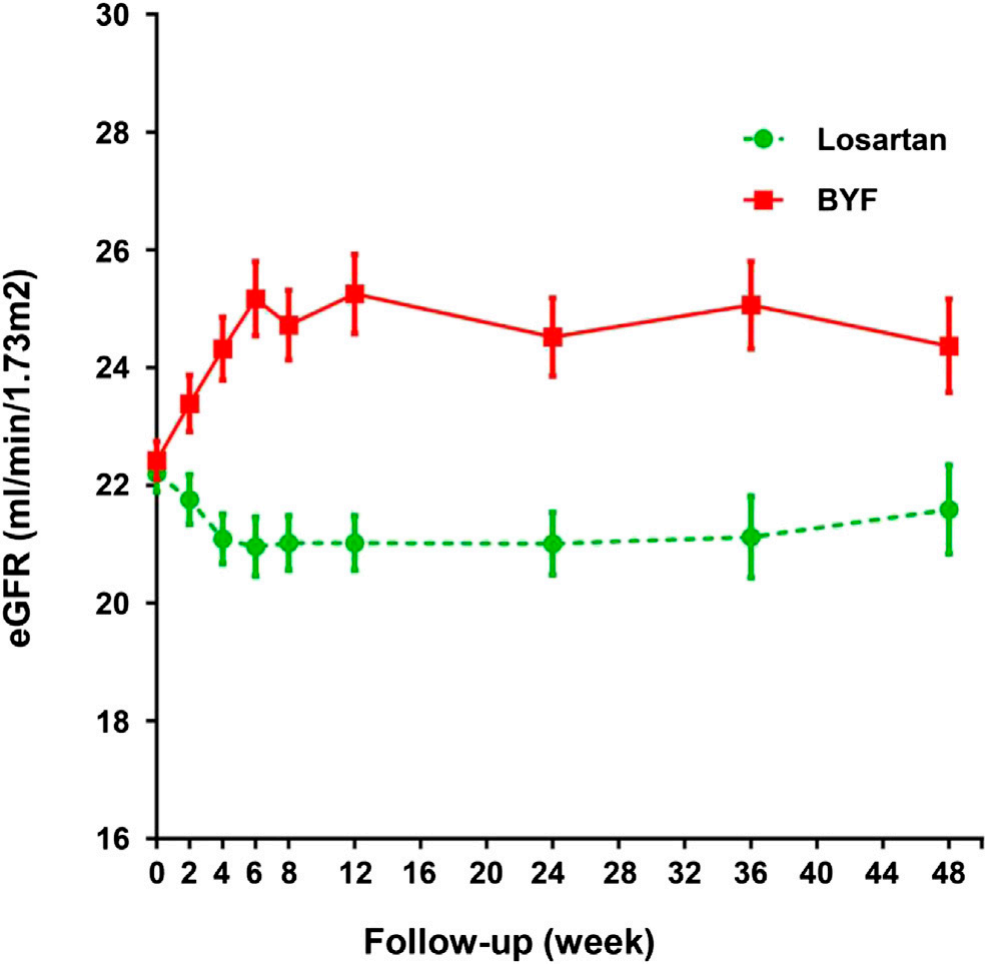
Changes in estimated glomerular filtration rate (eGFR) in the losartan and Bupi Yishen Formula (BYF) groups over 48 weeks.

In the FAS, the unadjusted mean slopes of eGFR were −4.53 (standard error [SE] 0.64) and −2.30 (SE 0.63) ml/min/1.73 m^2^/year in the losartan and BYF groups, respectively. The difference of adjusted mean slopes of eGFR between the two groups was −2.24 ml/min/1.73 m^2^ (95% confidence interval [CI]: −4.01,−0.46; *p*=0.014) over the 48 week interval (Table 2). As shown in Figure 2, the difference of mean slopes of eGFR between the two groups was about 4.23 ml/min/1.73 m^2^ prior to 12 weeks, and evidently attenuated to less than 3 ml/min/1.73 m^2^ post to 12 weeks (Supplementary Table S4). Correspondingly, the difference of unadjusted mean slopes of eGFR between the two groups was -2.25 ml/min/1.73 m^2^ (95%CI: −4.01,−0.46; *p*=0.013) over the first 12 weeks.

**TABLE 2:** Results of primary and composite outcome according to the study group (FAS population).

Variable	Losartan group (n=284)	BYF group (n=283)	Difference (95% CI)	P
**Primary outcome**				
Unadjusted eGFR slope (SE)^a^	−4.53 (0.64)	−2.30 (0.63)	−2.24 (−4.01, −0.46)	0.014
**Secondary outcomes**				
Composite endpoint^#^	95 (36.4%)	73 (28.1%)	0.61 (0.44, 0.85) ^b^	0.003
ESKD^#^	91 (34.9%)	71 (27.3%)	0.61 (0.43, 0.85)^b^	0.004
Stroke or cardiovascular events	3 (1.1%)	1 (0.4%)		-
Death	0	1 (0.4%)		-
Doubling of serum creatinine	1 (0.4%)	0		-

^a^ eGFR slope calculated in the mixed-effects model, no adjustment.# 18 cases without available data and 28 ineligible cases (ESKD at baseline) were excluded from analysis (23 in the losartan group and 23 in the BYF group).

^b^ Shown as Cox regression analysis results, HR (95%CI), adjusted center effect, sex, age, weight, systolic pressure, hypertension history and gout/hyperuricemia history.

BYF, Bupi Yishen Formula; CI, confidence interval; SE, standard error; eGFR, estimated glomerular filtration rate; ESKD, end-stage kidney disease.

### Secondary Outcomes

The composite endpoint was achieved by 73 (28.1%) participants in the BYF group and 95 (36.4%) participants in the losartan group. The corresponding figures for individual endpoints were 71 (27.3%) vs. 91 (34.9%) for ESKD, 0 (0%) vs. 1 (0.4%) for doubling of serum creatinine, 1 (0.4%) vs. 0 (0%) for death and 1 (0.4%) vs. 3 (1.1%) for stroke or cardiovascular events (Table 2).

**FIGURE 3:**
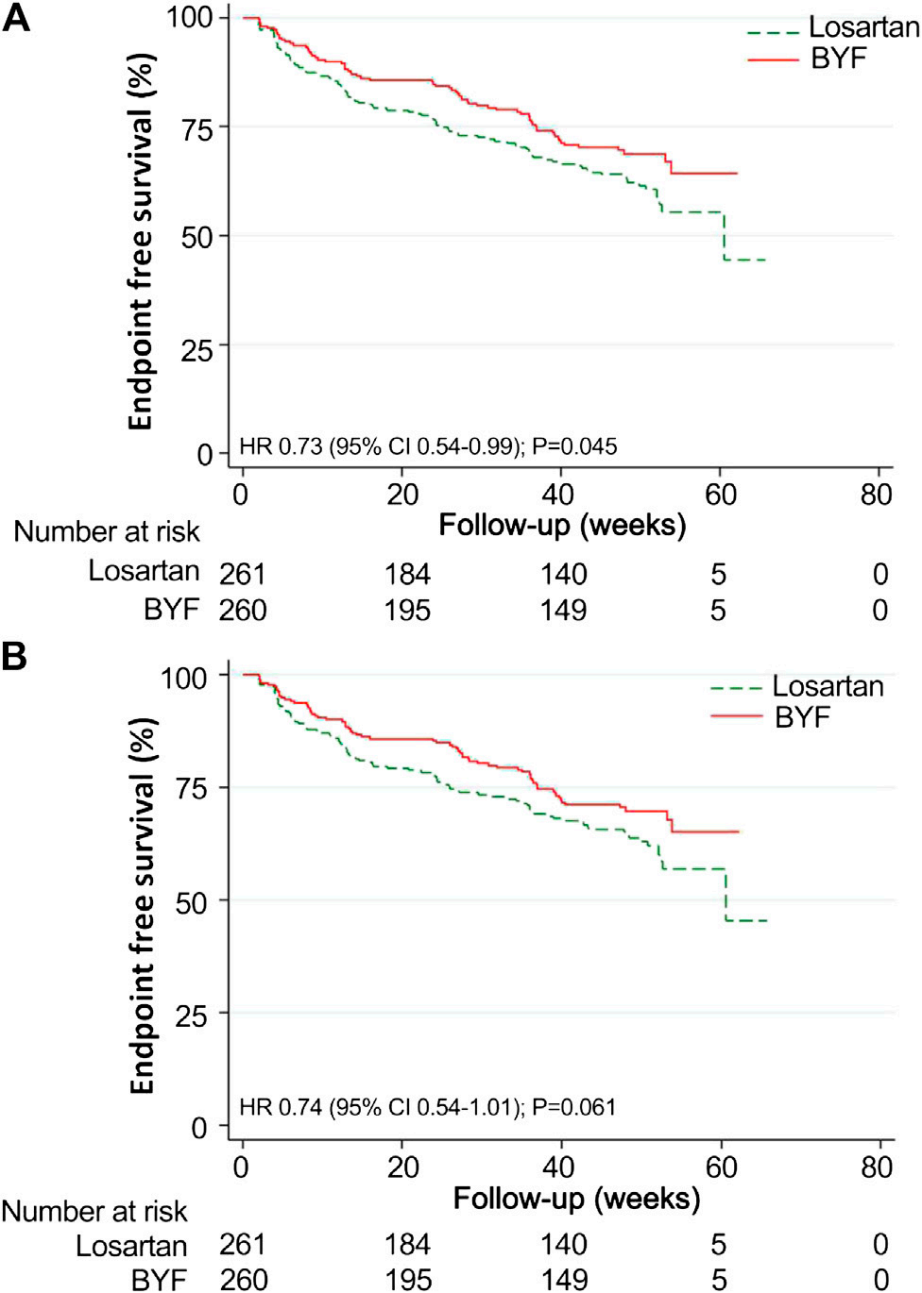
Kaplan-Meier survival curves for the Bupi Yishen Formula (BYF) and Losartan groups. Twenty-eight participants were excluded from the survival analyses as they reached end-stage kidney disease (ESKD) after randomization but before the initiation of treatment. **(A)** Composite endpoint (composite of death, doubled serum creatinine, ESKD, and cardiovascular or cerebrovascular events). **(B)** ESKD.

The mean time to the composite endpoint was significantly longer in the BYF group (49.3 weeks, 95%CI: 46.8,51.9) than in the losartan group (46.8 weeks, 95%CI: 43.6,49.9, *p* = 0.044) (Figure 3). Survival analysis suggested a 39% lower risk of composite endpoints in the BYF group compared with the losartan group (hazard ratio [HR]=0.73, 95%CI: 0.54,0.99, *p* = 0.045), consistent with the adjusted HR of the Cox model (HR=0.61, 95%CI: 0.44,0.85, *p* = 0.003). Most of the composite events were dominated by entering ESKD, and the adjusted risk of ESKD was significantly lower in the BYF group (HR=0.61, 95%CI: 0.43,0.85, *p* = 0.004). There were no between-group differences with respect to the other endpoints.

**FIGURE 4:**
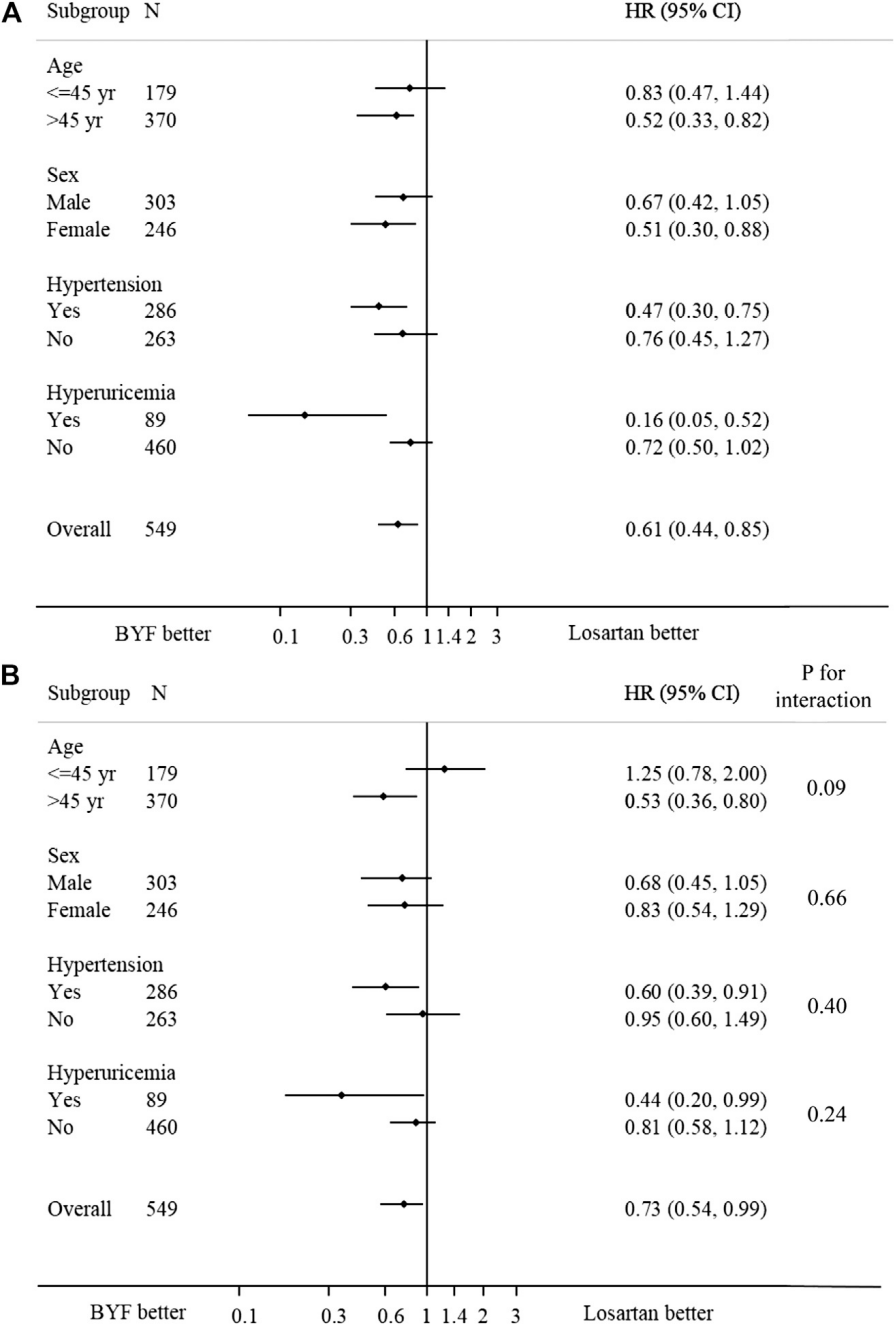
Forest plot of the effect of Bupi Yishen Formula (BYF) on the composite endpoint by subgroups. **(A)** Model 1: no adjustment. **(B)** Model 2: adjusted for center effect, sex, age, change of weight over 48 weeks, baseline estimated glomerular filtration rate (eGFR), and morbidities (hypertension, gout, or hyperuricemia).

In the subgroup analyses, no statistically significant interactions were found between the intervention and any of the subgroups including age, sex, hypertension and hyperuricemia (Figure 4).

Compared with baseline, urinary protein-creatinine-ratio decreased by 0.76 mg/mg in the losartan group and 0.34 mg/mg in the BYF group (*p* > 0.05). The serum creatinine reduced in the BYF group but increased in the losartan group (*p* = 0.041). Over the course of the study, there were rising trends of total serum cholesterol, low-density lipoprotein, and phosphate levels in the losartan group, in contrast to declines in the BYF group. The body weight tended to decrease in the losartan group but remained stable in the BYF group. There were no significant between-group differences observed in any other laboratory parameters (Supplementary Table S5,S6).

### Safety Evaluation

The frequencies of AEs, serious AEs, and AEs leading to withdrawal were similar in the two groups (Table 3). Hyperkalemia was the most common AE reported and was less commonly observed in the BYF group than in the losartan group (rate ratio 0.83, 95%CI: 0.67,1.03). Serious AEs leading to hospitalization occurred in six patients in the BYF group vs. ten in the losartan group. Of note, two cases of death occurred during follow-up: one patient in the BYF group died from an uncertain cause, and one patient in the losartan group died from gastrointestinal bleeding.

**TABLE 3:** Adverse events and serious adverse events according to the study group.

	BYF group (*n* = 276)		Losartan group (*n* = 278)		Rate ratio (95%CI)
	*n*	Rate per 100 patient-year	*n*	Rate per 100 patient-year	
Total AE^a^	272	148.9	281	161.8	0.92 (0.78, 1.09)
Hyperkalemia	164	89.8	188	108.3	0.83 (0.67, 1.03)
All SAE^a^,^b^	7	3.83	11	6.33	0.61 (0.20, 1.71)
AE leading to withdrawal	13	-	15	-	-
Neoplasms benign, malignant, and unspecified	0	-	2	1.15	-
Hospitalization for endometrial adenocarcinoma	0		1		
Hospitalization for lung cancer	0		1		
Cardiac disorders	1	0.55	1	0.58	0.95 (0.01, 74.64)
Acute myocardial infarction	1		1		
Hepatobiliary disorders	1	0.55	0	-	-
Hospitalization for liver function disorder	1				
Nervous system disorders	0	-	3	1.73	-
Hospitalization for transient ischemic attack	0		1		
Hospitalization for ischemic stroke	0		1		
Hospitalization for hemorrhagic stroke	0		1		
Musculoskeletal and connective tissue disorders	1	0.55	0	-	-
Hospitalization for gout	1				
Blood and lymphatic system disorders	1	0.55	2	1.15	0.48 (0.01, 9.13)
Hospitalization for decreasing white blood cell counts	1		2		
Gastrointestinal disorders	2	1.1	1	0.58	1.90 (0.10, 112.2)
Hospitalization for peptic ulcer	1		0		
Hospitalization for erosive gastritis	1		0		
Death due to gastrointestinal bleeding	0		1		
Metabolism and nutrition disorders	0	-	1	0.58	-
Hospitalization for hyperkalemia			1		
Infections and infestations	0	-	1	0.58	-
Hospitalization for pneumonia					
Other	1	0.55	0	-	-
Death due to unknown causes	1	0.55	0	-	-

^a^ Adverse events (AEs) and serious AEs (SAEs) were classified according to the Medical Dictionary for Regulatory Activities (MedDRA) central coding dictionary, Chinese version 20.0.

^b^ A serious adverse event was defined as death, a life-threatening episode, hospitalization, or prolongation of existing hospitalization, a persistent or substantial disability or incapacity, or an event otherwise considered to be an important medical event.

BYF, Bupi Yishen Formula; CI, confidence interval.

### Sensitivity Analysis

The results showed that eGFR at 48 weeks adjusted for baseline eGFR was higher in the BYF group (*p* < 0.0001). Similarly, decline in the adjusted eGFR slope using the mixed-effects model was comparably attenuated in BYF group (*p* = 0.013, Supplementary Table S7). To examine the potential impact of a possible early acute effect of therapy on renal function with respect to the overall result, sensitivity analyses were conducted. Thirty-three (12%) patients in the losartan group and thirteen (4.8%) patients in the BYF group experienced a serum creatinine increase of more than 30% in the first 12 weeks (*p* = 0.002). After excluding these patients, we observed a higher mean of eGFR at the end of follow-up and a slower decline of eGFR over 48 weeks in the BYF group (Supplementary Table S8), which remained consistent with that in FAS population. Lower risks of the composite endpoint and ESKD were still found in the BYF group (Supplementary Table S9). The results of the sensitivity analyses remained robust after excluding patients who took keto-amino acids during the trial and employing multiple imputation methods to deal with missing data (Supplementary Table S10–S13).

## Discussion

Our results demonstrated that, compared with losartan, BYF (the main of components were listed in Supplementary Table S2) resulted in a significantly slower decline of eGFR, particularly in the first 12 weeks, and lower risks of the composite outcome and ESKD in non-diabetic CKD4 patients over 48 weeks of follow-up. These results remained robust to a series of sensitivity analyses. In the subgroup analyses, no statistically significant interactions were found between the intervention and any of the subgroups including age, sex, hypertension and hyperuricemia. Serious adverse events and adverse events leading to withdrawal were uncommon and the incidences were similar between the two groups.

The ACEi or ARB of conventional CKD strategies is administered to achieve optimal BP control and proteinuria reduction, factors possibly associated with retarded CKD progression and decreased mortality risk (Li et al., 2013; Bhandari et al., 2016). ACEi and ARB are thought to produce long-term renoprotection through reduction of intra-glomerular pressure, which also results in a reversible early drop in kidney function. However, these effects may be harmful in patients with advanced CKD with seriously compromised kidney function, and has led to ongoing, multi-centre, randomized controlled STOP-ACEi trial which is assessing the effects of withdrawal of ACEi/ARB treatment in 410 CKD4-5 patients (Bhandari et al., 2016). Since the use of ACEi or ARBs in the CKD4-5 population is controversial and associated with a higher risk of adverse events(e.g. hyperkalaemia and acute kidney injury) (Strippoli et al., 2006; Bhandari et al., 2016), the presented study attempted to determine if there was a safe, more effective alternative in patients with stage 4 CKD using BYF. We found that, compared with the ARB losartan, BYF had favourable effects on kidney function decline and resulted in a 39% reduction in the composite outcome of death from any cause, doubling of serum creatinine level, end-stage kidney disease (ESKD), stroke, or cardiovascular events.

Comparable beneficial renal outcomes have also been previously reported for CHM products administered to patients with advanced CKD. Further study of the same cohort found that use of certain herbs contained within BYF, such as Astragalus membranaceus and Salvia miltiorrhiza, were associated with a lower risk of all-cause mortality (Hsieh et al., 2017). A systematic review of 22 studies including more than one thousand CKD participants supported the use of Astragalus preparation (the main herb of BYF) in reducing serum creatinine and urinary protein excretion, and improving creatinine clearance, though the quality of evidence was low (Zhang et al., 2014). Subgroup analysis found that the favourable effect of Astragalus preparation on serum creatinine was particularly evident in patients with later stage CKD.

Due to the multi-ingredients nature of the CHM formula, the exact mechanism of action and targets of BYF in patients with CKD are difficult to fully elucidate. In previous studies by our group, ultra-high performance liquid chromatography-mass spectrometry was used to identify compounds contained in BYF, and 86 compounds were detected with seven representative constituents (Zhang et al., 2018b). Pharmacological experiments with selected chemicals suggested that the renoprotective effects of BYF were probably mediated via anti-inflammatory, antioxidant, anti-fibrotic pathways, and gut microbiota modulatory effects (Supplementary Table S3) (Zhang et al., 2012b; Wang et al., 2014; Zhang et al., 2018). In addition, renal ischemia-reperfusion-induced injury can be attenuated by some of the compounds (Zhang et al., 2012b). Further studies are still needed to clarify the mechanism of action and related targets of BYF in CKD.

The beneficial effect of BYF on kidney function was observed early in the first 12 weeks and was sustained thereafter. It is conceivable that BYF may have early haemodynamic effects that contributed to this observation given that the BYF components of Astragalus, Salvia extract, Atractylodes, Dioscoreaop and Cuscuta have been shown to attenuate pulmonary arterial hypertension (Yuan et al., 2017), lower left ventricular end-diastolic pressure (Wang et al., 2017), inhibit the activation of rennin-angiotensin-aldosterone system(RAAS) (Cui et al., 2018), reduce glomerular volume (Guo et al., 2016), and decrease BP respectively (Patel et al., 2012). Variability in glomerular haemodynamics is a crucial prognostic risk factor for renal endpoints (Tsai et al., 2019). Nevertheless, most subjects recruited in our trial experienced little BP fluctuation to achieve BP goal after enrollment due to their intensive BP control management, and we did not obtain relevant vasodilatory cues for further investigation. Further study of intrarenal hemodynamic measurements are warranted in future studies.

The herbal ingredients of BYF were free from aristolochic acid, and hyperkalaemia was less common in the BYF group than in the losartan group. BYF appeared to be well tolerated among participants and no safety signals were observed. The result of our study suggested that not all herbal remedies were harmful to the kidney and that some, such as BYF, were beneficial to the kidney when used under the guidance of Chinese medicine professionals. As complementary and alternative medicines, including CHM, are a potentially important and inexpensive means of meeting kidney health care needs globally, the rigorous evaluation of therapy regarding complementary and alternative medicine, such as occurred in this RCT, is fundamental and meaningful, especially for low and middle-income countries.

This study was designed as a double-dummy, double-blind RCT, with strict quality control processes for sourcing and manufacturing BYF and matched identical placebo. These strengths should be balanced against the study’s limitations. Firstly, the clinical utility of serum creatinine-based eGFR may have been influenced by body mass, diet and creatinine measurement techniques. To address this issue, our results remained robust after adjustment for change of body weight over 12 months. Secondly, since our study duration was relatively short (48 weeks), further investigation with longer follow-up is warranted to validate the impact of BYF in terms of eGFR trajectory and important clinical outcomes. Thirdly, patients who reached the composite end-point (36.4% in Losartan group and 28.1% in BYF group, *p* = 0.003), were not continued to be followed, which may have introduced informative censoring and contributed to missing data during the later follow up. The mixed-effects model was used in this study, as recommended in Karen et al.’s paper (Leffondre et al., 2015), to make the results robust. Fourthly, urinary creatinine(UCr) and creatinine clearance (CCr) measurements were not available. Finally, only patients with stage 4 CKD were recruited into the study, such that the results may not have been generalizable to patients with earlier stage CKD.

## Conclusion

BYF might have renoprotective effects among non-diabetic patients with CKD4 in the first 12 weeks and over 48 weeks, but longer follow-up is required to evaluate the long-term effects. This could represent an alternative for patients reluctant to take renin-angiotensin blocking medications or in low-income settings.

## Data Availability

Data cannot be made publicly available as the dataset contains sensitive and identifying information. The authors confirm that the data will be made available upon request. Requests should be sent to both two corresponding authors, Zehuai Wen (wenzh@gzucm.edu.cn) and Xusheng Liu (liuxusheng@gzucm.edu.cn).
