# Supplementary material for: Bupi Yishen Formula Versus Losartan for Non-Diabetic Stage 4 Chronic Kidney Disease: A Randomized Controlled Trial
Source: Front Pharmacol. 2021 Jan 29;11:627185. doi: 10.3389/fphar.2020.627185 (PMC7941267; doi:10.3389/fphar.2020.627185)
Supplement: Supplementary file 1 [file datasheet1.docx]

Supplementary Material

**Supplementary Table 1.** Trial sites and investigators.

|  | **Clinical Sites** | **Investigators** |
| --- | --- | --- |
| 01 | Guangdong Provincial Hospital of Chinese Medicine | Xu sheng Liu |
| 02 | First Teaching Hospital of Tianjin University of Traditional Chinese Medicine | Hong tao Yang |
| 03 | Longhua Hospital Affiliated to Shanghai University of Traditional Chinese Medicine | Yue yi Deng |
| 04 | Hangzhou Hospital of Traditional Chinese Medicine | Hong yu Chen |
| 05 | Jiangsu Province Hospital of Traditional Chinese Medicine | Wei Sun |
| 06 | Dognzhimen Hospital Beijing University of Traditional Chinese Medicine | Yu ning Liu  Jing Li |
| 07 | The First Affiliated Hospital of Heilongjiang University of Chinese Medicine | Li qun Song |
| 08 | Teaching Hospital of Chengdu University of Traditional Chinese Medicine | Ming Chen |
| 09 | Shanxi Province Hospital of Traditional Chinese Medicine | Xiao hong Cheng  Yun Tian |
| 10 | Yunnan Province Hospital of Traditional Chinese Medicine | Chun yan Zhang |
| 11 | Shenzhen Traditional Chinese Medicine Hospital | Shun min Li  Guo liang Xiong |
| 12 | The First Affiliated Hospital of Guangxi University of Traditional Chinese Medicine | Wei Shi |
| 13 | The First Affiliated Hospital of Guiyang College of Traditional Chinese Medicine | Ji hong Zhan |
| 14 | The Affiliated Hospital of Liaoning University of Chinese Medicine | Fang Yuan |
| 15 | The First Affiliated Hospital of Guangdong University of Chinese Medicine | Shui fu Tang Gang yi Chen |
| 16 | Liuzhou Hospital of Traditional Chinese Medicine | Liang Li Jian guo Guan |
| 17 | Xinhui Hospital of Traditional Chinese Medicine | Feng Lin |
| 18 | Guangzhou Hospital of Traditional Chinese Medicine | Jiazhen Rao |
| 19 | The First Hospital of Shanxi Medicine University | Jing ai Fang |
| 20 | Hubei Province Hospital of Traditional Chinese Medicine | Xiao qin Wang |
| 21 | The Affiliated Jiang men Traditional Chinese Medicine Hospital, Jinan University | Ai cheng Yang |

### Supplementary Table 2. The components of BupiYishen Formula in Chinese, English, and Latin names, medicinal parts, and place of origin.

| **Chinese Pin Yin name** | **Latin binomial name** | **English name** | **Medicinal parts** | **Place of origin (Province)** | **Ratio** |
| --- | --- | --- | --- | --- | --- |
| Huang qi | *Astragali radix* | *Astragalus membranaceus* | Root | Neimenggu, Gansu | 6 |
| Dang shen | *Codonopsitis radix* | *Codonopsis pilosula* | Root | Gansu | 4 |
| Bai zhu | *Atractylodis macrocephalae rhizoma* | *Atractylodes*  *macrocephala* | Stem | Zhejiang, Anhui | 3 |
| Fu ling | *Poria* | *Poriacocos* | Sclerotium | Hu’nan | 3 |
| Shan yao | *Diosscoreae rhizoma* | *Dioscoreaop posita* | Root | He’nan | 3 |
| Yi yi ren | *Coicis semen* | *Coix seed* | Seed | Guizhou | 4 |
| He shou wu | *Polygoni multiflori radix* | *Polygonum multiflorum* | Root | He’nan | 3 |
| Tu si zi | *Cuscutae semen* | *Cuscuta Chinensis* | Seed | Shandong | 3 |
| Dan shen | *Salviae miltiorrhizae radix et rhizoma* | *Salvia miltiorrhiza* | Root | Anhui, Jiangsu | 3 |

**Supplementary Table 3.** Main chemical components of BupiYishen Formula and possible pharmacological effects (identified by liquid chromatography-mass spectrometry).

| **English name** | **Chemical Compounds** | **Possible renal protective effects** |
| --- | --- | --- |
| *Astragalus membranaceus* | Calycosin-7-O-β-D-glup, Odoratin-glup, Pratensein-7-O-Glu, Formononetin-7-O-Glc, 6,4’-Dimethoxyisoflavone-7-O-Glc, Calycosin-7-O-glc-6’’-Oacetate, 9,10-Dimethoxypterocarpan-3-O-β-D-glucopyranoside, Calycosin, Isomucronulatol-7-O-β-Dglucopyranoside, 7-Hydroxy-6,4’-dimethoxyisoflavone, Formononetin-7-O-β-Dglycoside-6’’-O-acetate, Rhamnocitrin, 9,10-Dimethoxypterocarpan-3-O-β-D-glucopyranosideacetate, Astragaloside I - III , V-VII, Formononetin, Soyasaponin I, Isoastragaloside I -II, Acetylastragaloside I | anti-inflammatory,  anti-oxidant,  anti-fibrotic, improvement incidence of infection |
| *Codonopsispilosula* | Tangshenoside V, Azelaic acid, Lobetyolin, 6’’’-Trans-p-coumaroyltangshenoside I, 6’’’-Cis-p-toumaroyltangshenoside I, 9,12,13-Trihydroxyoctadec-10,15-dienoic acid, 9,12,13-Trihydroxyoctadec-10-enoic acid, 5,6,9-Trihydroxy-octadec-7-enoic acid | immunological adjuvant,  nutritional status improvement, anti-oxidant |
| *Atractylodes*  *macrocephala* | Atractylenolide I, Atractylenolide II | nutritional status improvement,anti-oxidant |
| *Poriacocos* | Pachymaran, Citric acid | immunological adjuvant |
| *Dioscoreaopposita* | Dioscin | anti-inflammatory,  anti-oxidant |
| *Coix seed* | Azelaic acid, 5,6,9-Trihydroxy-octadec-7-enoic acid, Coronaric acid | immunological adjuvant,  anti-fibrotic |
| *Polygonum multiflorum* | (Z)-THSG, (E)-THSG, 2,3,5,4′-Tetrahydroxystilbene-2- (galloyl)-O-glc, Emodin-8-O-glc, Physcion-8-O-glc, Emodin, | anti-oxidant |
| *Cuscutachinensis* | 3-Caffeoyl-quinic acid, 5-Caffeoyl-quinic acid, 4-Caffeoyl-quinic acid, Quercetin 3-O- (2-O-apisyl)-galactoside, Quercetin 3-O-galactoside (hyperoside), Astragalin, Cuscutamine, Kaempferol or luteolin, Cuscutic acid C/Isomer, Acely-cuscutic acid C | anti-oxidant |
| *Salvia miltiorrhiza* | Salvianolic acid D, Salvianolic acid H or I, Rosmarimic acid, Lithospermic acid, Salvianolic acid B, Salvianolic acid A, Salvianolic acid E, Monomethyl lithospermater, Isomer of Salvianolic acid A, Salvianolic acid C, 9,12,13-Trihydroxyoctadec-10,15-dienoic acid | anti-inflammatory,  anti-oxidant |

Abbreviation: pen, pentoside; glup, glucopyranoside.

**Supplementary Table 4.** Values of eGFR at different time points during the 48 weeks follow-up.

| Timepoints | Group | N | eGFR (ml/min/1.73 m^2^) | P |
| --- | --- | --- | --- | --- |
| Baseline | Losartan | 283 | 22.33±5.54 | 0.662 |
|  | BYF | 283 | 22.54±6.10 |  |
| 2 weeks | Losartan | 255 | 21.76±6.74 | 0.012 |
|  | BYF | 251 | 23.38±7.66 |  |
| 4weeks | Losartan | 255 | 21.04±6.68 | <0.001 |
|  | BYF | 254 | 24.28±8.42 |  |
| 6 weeks | Losartan | 200 | 21.01±7.06 | <0.001 |
|  | BYF | 215 | 25.19±9.22 |  |
| 8 weeks | Losartan | 237 | 21.02±7.03 | <0.001 |
|  | BYF | 235 | 24.72±9.01 |  |
| 12 weeks | Losartan | 230 | 21.05±6.90 | <0.001 |
|  | BYF | 226 | 25.28±10.09 |  |
| 24 weeks | Losartan | 187 | 21.04±7.15 | <0.001 |
|  | BYF | 197 | 24.58±9.26 |  |
| 36 weeks | Losartan | 159 | 21.12±8.68 | <0.001 |
|  | BYF | 165 | 24.98±9.54 |  |
| 48 weeks^a^ | Losartan | 143 | 21.62±8.97 | 0.013 |
|  | BYF | 147 | 24.36±9.60 |  |
| 48 weeks –baseline^a^ | Losartan | 143 | -1.70±7.63 | 0.003 |
|  | BYF | 147 | 1.04±7.88 |  |
| 48 weeks^c^ | Losartan | 118 | 21.97±9.09 | 0.075 |
|  | BYF | 119 | 24.02±8.61 |  |
| 48 weeks –baseline^c^ | Losartan | 119 | -1.43±7.96 | 0.033 |
|  | BYF | 119 | 0.76±7.73 |  |

Note: At baseline, one case was not included in the analysis due to patient withdrawal after randomization. a: Full-analysis set without dealing with missing data; c: Per-protocol set analysis without dealing with missing data.

eGFR: estimated glomerular filtration rate; BYF: BupiYishen Formula.

**Supplementary Table 5.** Laboratory parameters during the trial.

| **Laboratory parameter** | **Losartan ^a^** | | **BYF ^a^** | | **Difference between treatment groups ^b^ (95% CI)** | **P** |
| --- | --- | --- | --- | --- | --- | --- |
|  | **N** | **Mean±SD** | **N** | **Mean±SD** |  |  |
| **UPCR (mg/mg Cr)** | | | | | | |
| Baseline | 187 | 1.91±2.54 | 197 | 1.70±1.74 | 0.21 (-0.23,0.64) | 0.353 |
| Week 48 | 90 | 1.80±7.36 | 106 | 1.80±2.31 | 0.00 (-1.49,1.49) | 0.999 |
| Change from baseline | 77 | -0.76±8.06 | 96 | -0.34±2.67 | 0.41 (-2.14,1.32) | 0.637 |
| **Serum creatinine (μmol/L)** | | | | | | |
| Baseline | 282 | 253.51±55.83 | 281 | 258.05±76.05 | -4.55 (-15.59,6.49) | 0.419 |
| Week 48 | 143 | 275.66±93.78 | 147 | 253.14±92.85 | 22.52 (0.95,44.09) | 0.041 |
| Change from baseline | 142 | -34.07±75.47 | 146 | -11.98±80.62 | -22.08 (-40.21,-3.96) | 0.017 |
| **BUN (mmol/L)** | | | | | | |
| Baseline | 282 | 13.81±4.00 | 280 | 13.47±4.19 | 0.34 (-0.34,1.02) | 0.328 |
| Week 48 | 143 | 13.78±4.41 | 147 | 14.53±4.70 | -0.73 (-1.80,0.31) | 0.166 |
| Change from baseline | 142 | 0.47±3.77 | 145 | 1.20±3.92 | 0.74 (-0.16,1.63) | 0.105 |
| **iPTH (pmol/L)*** | | | | | | |
| Baseline | 240 | 17.75±15.85 | 244 | 15.64±12.44 | 2.11 (-0.43,4.65) | 0.104 |
| Week 48 | 115 | 19.08±15.15 | 125 | 20.29±19.34 | -1.20 (-5.65,3.24) | 0.594 |
| Change from baseline | 109 | -1.64±14.18 | 120 | -4.80±13.83 | 3.15 (-0.50,6.80) | 0.090 |
| **Serum albumin (g/L)*** | | | | | | |
| Baseline | 273 | 42.76±4.21 | 271 | 42.21±5.13 | 0.55 (-0.24,1.34) | 0.169 |
| Week 48 | 142 | 43.22±2.90 | 145 | 43.19±4.22 | 0.03 (-0.81,0.88) | 0.939 |
| Change from baseline | 136 | 0.12±3.48 | 139 | -0.16±4.07 | -0.28 (-0.62,1.1.8) | 0.540 |
| **Total cholesterol (mmol/L)*** | | | | | | |
| Baseline | 280 | 5.08±1.20 | 278 | 5.07±1.48 | 0.01 (-0.22,0.23) | 0.960 |
| Week 48 | 140 | 4.94±1.18 | 146 | 5.28±1.31 | -0.33 (-0.63,-0.04) | 0.024 |
| Change from baseline | 139 | 0.18±0.93 | 144 | -0.23±1.06 | 0.42 (0.18,0.65) | 0.001 |
| **Triglyceride (mmol/L)*** | | | | | | |
| Baseline | 280 | 1.89±1.21 | 277 | 2.06±1.55 | -0.18 (-0.41,0.05) | 0.133 |
| Week 48 | 140 | 1.93±1.80 | 146 | 2.03±1.49 | -0.10 (-0.48,0.29) | 0.620 |
| Change from baseline | 139 | -0.05±1.29 | 143 | 0.01±1.15 | -0.06 (-0.35,0.22) | 0.656 |
| **Low-density lipoprotein (mmol/L)*** | | | | | | |
| Baseline | 274 | 3.03±0.99 | 267 | 3.02±1.23 | 0.01 (-0.17,0.20) | 0.883 |
| Week 48 | 137 | 2.95±1.01 | 143 | 3.14±1.05 | -0.19 (-0.43,0.05) | 0.118 |
| Change from baseline | 131 | 0.16±0.74 | 135 | -0.23±0.95 | 0.39 (0.18,0.60) | <0.001 |
| **Serum calcium (mmol/L)^c^** | | | | | | |
| Baseline | 270 | 2.27±0.18 | 273 | 2.26±0.17 | 0.01 (-0.02,0.04) | 0.361 |
| Week 48 | 132 | 2.27±0.16 | 146 | 2.29±0.17 | -0.02 (-0.06,0.01) | 0.211 |
| Change from baseline | 125 | 0.04±0.18 | 142 | 0.00±0.21 | 0.03 (-0.01,0.08) | 0.149 |
| **Serum phosphate (mmol/L)^c^** | | | | | | |
| Baseline | 269 | 1.27±0.26 | 273 | 1.29±0.26 | -0.01 (-0.06,0.03) | 0.540 |
| Week 48 | 134 | 1.21±0.22 | 145 | 1.31±0.28 | -0.10 (-0.16,-0.04) | 0.001 |
| Change from baseline | 125 | 0.01±0.26 | 141 | -0.06±0.27 | 0.08 (0.01,0.14) | 0.021 |
| **SBP (mmHg)** | | | | | | |
| Baseline | 268 | 127.2±11.2 | 262 | 130.1±11.6 | -2.8 (-4.8,0.9) | 0.005 |
| Week 48 | 181 | 126.8±11.3 | 187 | 128.0±11.0 | -1.2 (-3.5,1.1) | 0.293 |
| Change from  baseline | 181 | 0.3±11.1 | 187 | -0.2±12.7 | 0.5 (-1.9,2.9) | 0.683 |
| **DBP (mmHg)** | | | | | | |
| Baseline | 268 | 78.9±8.5 | 262 | 80.7±8.6 | -1.9 (-3.3,-0.4) | 0.012 |
| Week 48 | 181 | 77.6±8.3 | 187 | 79.2±7.8 | -1.5 (-3.2,0.1) | 0.068 |
| Change from baseline | 181 | 1.7±8.8 | 187 | 0.3±9.0 | 1.4 (-0.4,3.2) | 0.128 |
| **Body weight (kg)** | | | | | | |
| Baseline | 284 | 61.74±11.56 | 283 | 61.69±11.11 | 0.05 (-1.82,1.92) | 0.959 |
| Week 48 | 146 | 62.43±12.46 | 150 | 60.89±10.24 | 1.55 (-1.06.4.15) | 0.244 |
| Change from baseline | 146 | -0.68±2.69 | 150 | 0.10±3.04 | -0.78 (-1.44,-0.12) | 0.020 |

a. Statistical significance for the within-group changes determined from a test comparing the mean changes to zero.

b. Statistical significance for the between-group changes determined from a test comparing the difference in mean changes between Losartan and BYF groups.

c. Rank-sum test.

BYF: BupiYishen Formula; SD: standard deviation; CI: confidence interval; UPCR: urinary protein/creatinine ratio; Cr: creatinine; BUN: blood urea nitrogen; iPTH: intact parathyroid hormone; SBP: systolic blood pressure; DBP: diastolic blood pressure.

### Supplementary Table 6. Changes in urinary protein/creatinine ratio (UPCR) in the losartan and BYF groups over 48 weeks.

###



BYF: Bupi Yishen Formula.

### Supplementary Table 7. Sensitivity analysis: adjusted eGFR slope calculated in the mixed-effects model using baseline eGFR, sex, age, mean change of weight, history of hypertension, and history of gout or hyperuricemia as covariates.

| **Variable** | **Losartan group (n=284)** | **BYF group (n=283)** | **Difference (95% CI)** | **P** |
| --- | --- | --- | --- | --- |
| **Primary outcome** | | | | |
| eGFR at 48 weeks (95%CI) adjusted for baseline eGFR* | 20.1 (19.1, 21.2) | 23.2 (22.2, 24.2) | -3.0 (-4.3, -1.8) | <0.0001 |
| Adjusted eGFR slope (SE)*^**^* | -4.54 (0.64) | -2.29 (0.64) | -2.25 (-4.03, -0.47) | 0.013 |

Note: * Shown as estimate marginal means adjusted for baseline eGFR. 18 cases without available follow up data were excluded from analysis (8 in losartan group and 10 in BYF group).

** eGFR slope calculated in the mixed-effects model, adjusted center effect, sex, age, weight, systolic pressure, hypertension history and gout/hyperuricemia history.

BYF: Bupi Yishen Formula.

### Supplementary Table 8. Sensitivity analysis: mean change of eGFR over 48 weeks excluding patients who experienced a creatinine increase ≥30% within the first 12 weeks.

| **Variable** | **Losartan group**  **(n=207)** | **BYF group**  **(n=218)** | **Difference**  **(95% CI)** | **P** |
| --- | --- | --- | --- | --- |
| **Primary outcome** | | | | |
| eGFR at wk 48  mean (95%CI )^a^ | 20.6 (19.5 to 21.7) | 23.5 (22.4 to 24.5) | -2.9 (-4.2 to -1.6) | <0.001 |
| eGFR slope (SE) ^b^ | -3.40 (0.63) | -1.45 (0.60) | -1.95 (-3.67, -0.23) | 0.026 |
| eGFR slope (SE) ^c^ | -3.39 (0.63) | -1.42 (0.60) | -1.96 (-3.68, -0.24) | 0.025 |

Note: There were 33 patients (12.0%) in the losartan group and 13 patients (4.8%) in the BYF group who experienced serum creatinine increased ≥30% within the first 12 weeks (p = 0.002). In addition, 18 cases were excluded from analysis due to no available data (8 cases in the losartan group, 10 cases in the BYF group).

a. Shown as mixed model adjusted mean, adjusted center effect, sex, age, weight, systolic pressure, hypertension history, and gout/hyperuricemia history;

b. eGFR slope calculated in the Mixed-effects model, no adjustment;

c. eGFR slope calculated in the Mixed-effects model, adjusted center effect, sex, age, weight, systolic pressure, hypertension history, and gout/hyperuricemia history

BYF: Bupi Yishen Formula.

### Supplementary Table 9. Sensitivity analysis: survival analysis (Cox regression) results of patients between groups excluding patients who experienced a creatinine increase ≥30% within the first 12 weeks.

| **Model** | **Events** | **HR (95%CI)**  **BYF vs. Losartan** | **P** |
| --- | --- | --- | --- |
| **Model 1** |  |  |  |
| **Composite endpoint** | 135 | 0.77 (0.55-1.08) | 0.133 |
| **ESRD** | 129 | 0.79 (0.56-1.19) | 0.184 |
| **Model 2** |  |  |  |
| **Composite endpoint** | 135 | 0.64 (0.44-0.93) | 0.019 |
| **ESRD** | 129 | 0.64 (0.43-0.94) | 0.023 |

Model 1: No adjustment

Model 2: Adjusted for center effect, gender, age, change of weight over 12 months, baseline eGFR, and morbidities (hypertension, gout, or hyperuricemia)

ESRD: end-stage renal disease; BYF: Bupi Yishen Formula.

.

### Supplementary Table 10. Sensitivity analysis: the change of eGFR over 48 weeks excluding patients who took keto-amino acids during the trial.

|  | **Group** | **N** | **Mean ± SD** | **Difference  (95%CI)** | **t** | **P** |
| --- | --- | --- | --- | --- | --- | --- |
| Baseline | Losartan | 251 | 22.14 ±5.24 | -0.34 (-1.25,0.58) | 0.718 | 0.473 |
|  | BYF | 258 | 22.47 ±5.31 |  |  |  |
| 48 weeks | Losartan | 134 | 21.51 ±9.07 | -2.99 (-5.24,-0.74) | 2.618 | 0.009 |
|  | BYF | 139 | 24.50 ±9.79 |  |  |  |
| 48 weeks-baseline ^a^ | Losartan | 134 | -1.62 ±7.83 | -2.83 (-4.71,-0.94) | 2.954 | 0.003 |
|  | BYF | 139 | 1.21 ±7.99 |  |  |  |

Note: 40 cases were excluded in this analysis (25 cases in the losartan group vs. 15 cases in the BYF group, P=0.108).

^a^ without dealing with missing data.

SD: standard deviation; BYF: Bupi Yishen Formula.

.

### Supplementary Table 11. Sensitivity analysis: mean change of eGFR over 48 weeks excluding patients who took keto-amino acids during the trial.

| **Variable** | **Losartan group**  **(n=284)** | **BYF group**  **(n=283)** | **Difference**  **(95% CI)** | **P** |
| --- | --- | --- | --- | --- |
| **Primary outcome** | | | | |
| eGFR at wk 48  mean (95%CI) ^a^ | 20.0 (19.0 to 21.1) | 23.2 (22.2 to 24.3) | -3.2 (-4.5 to -1.9) | <0.001 |
| eGFR slope (SE) ^b^ | -4.58 (0.68) | -2.22 (0.66) | -2.36 (-4.22,-0.50) | 0.013 |
| eGFR slope (SE) ^c^ | -4.58 (0.68) | -2.21 (0.66) | -2.37 (-4.23,-0.51) | 0.013 |

Note: 40 cases were excluded in this analysis (25 cases in the losartan group versus 15 cases in the BYF group, P=0.108). In addition, 18 cases were excluded from analysis due to no available data (8 cases in the losartan group, 10 cases in the BYF group).

a. Shown as Mixed model adjusted mean, adjusted center effect, sex, age, weight, systolic pressure, hypertension history, and gout/hyperuricemia history;

b. eGFR slope calculated in the Mixed-effects model, no adjustment;

c. eGFR slope calculated in the Mixed-effects model, adjusted center effect, sex, age, weight, systolic pressure, hypertension history, and gout/hyperuricemia history

BYF: Bupi Yishen Formula; CI: confidence interval; eGFR: estimated glomerular filtration rate.

### Supplementary Table 12. Sensitivity analysis: survival analysis (Cox regression) results of patients between groups excluding patients who took keto-amino acids during the trial.

| **Model** | **Events** | **HR (95%CI)**  **BYF vs. Losartan** | **P** |
| --- | --- | --- | --- |
| **Model 1** |  |  |  |
| **Composite endpoint** | 155 | 0.76 (0.55-1.04) | 0.088 |
| **ESRD** | 150 | 0.77 (0.55-1.06) | 0.102 |
| **Model 2** |  |  |  |
| **Composite endpoint** | 155 | 0.66 (0.48-0.94) | 0.017 |
| **ESRD** | 150 | 0.66 (0.46-0.93) | 0.018 |

Model 1: No adjustment

Model 2: Adjusted for center effect, gender, age, change of weight over 12 months, systolic pressure, baseline eGFR and morbidities (hypertension, gout or hyperuricemia)

BYF: Bupi Yishen Formula; CI: confidence interval; ESRD: end-stage renal disease.

### Supplementary Table 13. Sensitivity analysis: the mean change of eGFR over 48 weeks between groups when using different methods to deal with the missing data

|  | **Complete cases** | **LOCF** | **MI** |
| --- | --- | --- | --- |
| **Mean change of baseline** | -2.72 | -2.79 | -2.94 |
| **95%CI** | -4.52，-0.92 | -4.11，-1.46 | -4.60,-1.28 |
| **SE** | 0.91 | 0.67 | 0.84 |
| **P** | 0.003 | 0.001 | 0.001 |

LOCF: last observation carried forward; MI: multiple imputations; CI: confidence interval; SE: standard error.
